# A Manual of Experiments Illustrative of Chemical Science

**Published:** 1833-10

**Authors:** 


*A Manual of Experiments illustrative of Chemical Science, systematically arranged: Remarks on the Nomenclature, &c.* By John Murray, F.S.A., &c. *Third Edition.* London, 1833. 12mo. pp. 149.

Mr. Murray, who is not only an F.S.A., an F.L.S., an F.H.S., and an F.G.S., but a member of nine mechanics' institutes, is decidedly one of the greatest little-book makers of the age; and we venture to prophesy that he will run Mr. Pinnock and Professor Rennie very hard. The public are busily engaged in reading fifteen treatises which he has already published, and two more are announced as in the press: nay, perhaps while we are penning these lines, the Essays on the Physiology of Plants, and on the History and Phenomena of Poisons, may have seen the light; for the utero-gestation of these tiny treatises is short, and the parturition easy. We are afraid, however, that we shall be considered as exhibiting the callousness of old and hardened reviewers, when we confess that we have never read any of these Lilliputian encyclopædias of every thing worth knowing; nay, that we have never read even Mr. Murray's Treatise on Pulmonary Consumption, though recommended by the Record, the Friend's Magazine, the Imperial Magazine, and other highly scientific authorities. This book, of which the Metropolitan Magazine said, on the 1st of February, 1832, that "the faculty cannot but highly appreciate Mr. Murray's volume," has never been appreciated by us at all; so that, in sitting down to the thin duodecimo before us, we do so with the glaring disadvantage of being unskilled in Murrayan ways of thinking.

Mr. Murray commences his manual with a panegyric on the modern chemical nomenclature, and says,

"A proper estimate of the superior value of the new nomenclature may be best obtained by comparison, contrasting the new and old in juxta position; and we much mistake, if, while it throws the old terms into the background and the shade, it does not bespeak a ready acquiescence in favour of the new nomenclature. In this estimate and contrast, amplification would be useless and uncalled for; the selection may therefore be limited, and yet supply an ample specimen. Oil of tartar, oil of vitriol, butter of antimony, horn silver, sugar of lead, and cream of tartar, are terms altogether void of meaning, and 'signify nothing.' The nomenclature, which forms a part of this volume, will supply abundant materials of a similar complexion. Is sugar of lead said to be descriptive of its peculiar sweetness? so are the salts of ittria and glaucina in a still higher degree. Oil of vitriol, &c. mislead by the adjunct oil, as the chemical constituents of oil are entirely absent. In the term copperas we consider copper to be present; and we naturally enough expect to find lead in 'black lead;' while the former is a sulphate of iron, and the latter a compound of iron and carbon. Nor is this the worst of these antiquated and unmeaning epithets, for the unwary would little suspect a fatal poison under the gifted name of 'acid of sugar."

"When we turn to the new nomenclature, a more welcome language presents itself, though it cannot be reasonably expected, that we are enabled to apply terms critically descriptive of some invariable feature, to all the principles and elements of chemical research. Could this indeed be effected, the structure erected would be a durable monument of skill, it would be stamped with a permanence which nothing could by possibility destroy, and the novelties of discovery could never affect. Chlorine and iodine are examples of this description, the names are full of meaning, and the features on which they are founded can never change. Chlorine as chlorine, whether simple as now considered, or hereafter proved to be compound, can never cease to be compound in a *green* attire; and iodine in the state of vapour will ever assume a violet colour. Chlorine is derived from a Greek word signifying green; and iodine from a Greek word importing violet. So far these names are expressive and appropriate." (P. 4.)

Unquestionably Mr. Murray must have been half asleep, or writing two little books at once, when he ventured to assert that "oil of tartar, &c." are terms altogether void of meaning. Cannot Mr. M. see, too, that if *sugar* of lead is a bad name, because the salts of ittria and glaucina are sweet, then *acetate* of lead is equally bad, since there are other acetates in abundance? Again, "oil of vitriol, &c." do *not* mislead by the adjunct *oil:* everybody knows that the expression is founded on one or two points of resemblance only. Suppose, for example, that some rival chemist, stung into envious madness by the never-ending praises bestowed on our author in the Courier, the Spectator, the Athenæum, the Bristol Mercury, &c., were to call him A GOOSE, surely he would not attempt to defend himself by observing, that he had no feathers, that he was not web-footed, that he was rarely, if ever, afflicted with the *cutis anserina,* and that his brother chemist was endeavouring to mislead the public by this "adjunct?" No; after retaliating in the most approved fashion, he would point to the fifteen published treatises, and the two with which he is big, and he would ask, are these the works of a goose?

Mr. Murray, though he has hit upon several imaginary advantages of the new nomenclature, has hardly hinted at the real one, namely, that it shows the proximate composition of the substances named.

It is scarcely worth our while to go through Mr. M.'s book. The experiments are of an amusing kind, and, as our author has a salutary horror of blowing up his readers, it may be safely recommended to persons in search of a little semi-scientific diversion: chemical students must get books of a very different kind.

